# Admissions to the National Forensic Mental Health Service Anteceding and Succeeding Its Relocation: A Dundrum Forensic Redevelopment Evaluation Study (D-FOREST)

**DOI:** 10.1192/bjo.2024.172

**Published:** 2024-08-01

**Authors:** Muhammad Umer Iqbal, M. Umer Waqar, Harry G Kennedy, Mary Davoren

**Affiliations:** 1 Dundrum Centre for Forensic Excellence, Trinity College Dublin, Dublin, Ireland; 2 Central Mental Hospital, National Forensic Mental Health Service, Portrane, Ireland

## Abstract

**Aims:**

The Central Mental Hospital is the Republic of Ireland's only secure forensic hospital and the seat of its National Forensic Mental Health Service (NFMHS). We scrutinised admission patterns in the NFMHS during the period 01/01/2018–01/10/2023; before and after relocating from the historic 1850 site in Dundrum to a modern facility in Portrane on 13/11/2022.

**Methods:**

This prospective longitudinal cohort study included all patients admitted during the above period. The study initially commenced in Dundrum and continued afterwards in Portrane. Data gathered included demographics, diagnoses, capacity to consent to treatment, and the need for intramuscular medication (IM) after admission. Therapeutic security needs and urgency of need for admission were collated from DUNDRUM-1 and DUNDRUM-2 scores rated pre-admission. Hours spent in seclusion during the first day, week, and month after admission were calculated. Data were collected as part of the Dundrum Forensic Redevelopment Evaluation Study (D-FOREST).

**Results:**

There were 117 admissions during the 69-month period. The majority were male (*n* = 98). Most were admitted from prisons (87%). Schizophrenia was the most common diagnosis (55.8%). Mean DUNDRUM-1 triage security scores were in the medium-security range (2.84–3.15) during this period. At the time of admission, 53.8% required seclusion, 25.6% required IM medication, and 79.5% lacked capacity to consent to treatment. Those who required seclusion on admission had worse scores on the DUNDRUM-2 triage urgency scale (*F* = 20.9, *p* < 0.001). On linear logistic regression, the most parsimonious model resolved with five predictors of hours in seclusion during the first day and week, which were: D1 item 8 – Victim sensitivity/public confidence issues, D1 item 10 – Institutional behaviour, D2 item 2 – Mental health, D2 item 4 – Humanitarian, and D2 item 6 – Legal urgency. 50% required IM medication during their first week of admission and these patients had significantly worse scores on: D1 item 8 – Victim sensitivity/public confidence issues, D1 item 10 – Institutional behaviour, D2 item 2 – Mental health, and D2 item 4 – Humanitarian (all *p* < 0.05).

**Conclusion:**

There was an increase in the frequency of admissions since relocating to Portrane. The results suggest that there was no change in overall triage security and urgency needs during the time period in question. Major mental illness related factors impacted the need for seclusion early in the admission, whereas factors linked to prison behaviour or personality-related factors were more associated with an ongoing need for seclusion at month one.

